# The role of impulsivity in the relationship between affect and alcohol consumption in young adults

**DOI:** 10.1111/acer.15192

**Published:** 2023-09-24

**Authors:** Gezelle Dali, Warren Logge, Benjamin Riordan, Tamlin S. Conner, Victoria Manning, E. Zayra Millan, Gavan P. McNally, Paul S. Haber, Kirsten C. Morley

**Affiliations:** ^1^ Specialty of Addiction Medicine, Sydney Medical School, Faculty of Medicine and Health University of Sydney Sydney New South Wales Australia; ^2^ Edith Collins Centre for Translational Research (Alcohol, Drugs & Toxicology) Royal Prince Alfred Hospital, Sydney Local Health District Sydney New South Wales Australia; ^3^ Centre for Alcohol Policy Research La Trobe University Melbourne Victoria Australia; ^4^ Department of Psychology University of Otago Dunedin New Zealand; ^5^ Monash Addiction Research Centre, Eastern Health Clinical School, Faculty of Medicine, Nursing & Health Sciences Monash University Melbourne Victoria Australia; ^6^ Turning Point, Eastern Health Richmond Victoria Australia; ^7^ School of Psychology University of New South Wales Sydney New South Wales Australia

**Keywords:** affect, alcohol use, ecological momentary assessment, impulsivity, young adults

## Abstract

**Background:**

Theoretical models of alcohol use posit that individuals consume alcohol to ameliorate negative affect or to heighten positive affect. It is important, however, to consider the influence of factors that may determine an individual's tendency to consume excessive amounts of alcohol under positive and negative circumstances. Thus, the current study examined a large sample of young adults to clarify whether positive and negative affect predict total alcohol consumption on drinking days and whether facets of impulsivity moderate these relationships.

**Methods:**

Six‐hundred ninety‐three young adults (*M*_age_ = 19.71 years, SD = 2.04; female = 62.9%) completed the Behavioral Inhibition System/Behavioral Activation System (BIS/BAS) scales at baseline followed by daily measures of positive and negative affect and self‐reported alcohol use for 13 days. Generalized linear mixed models were specified to assess the role of pre‐consumption affect on total drinks consumed across drinking days and to determine the moderating effect of each BIS/BAS subscale.

**Results:**

Participants were significantly more likely to drink in greater quantities on occasions preceded by higher positive affect but not negative affect. While fun‐seeking positively predicted total drinks consumed, there were no significant interaction effects between the BIS/BAS subscales and affect on total drinks consumed.

**Conclusions:**

These findings challenge existing affect regulation models and have implications for ecological momentary interventions aimed at addressing hazardous drinking behaviors.

## Graphical abstract

This ecological momentary assessment study examined whether positive and negative affect predict alcohol consumption and whether facets of impulsivity moderate these relationships in young adults. Participants consumed greater quantities on occasions preceded by higher positive affect but not negative affect. Fun‐seeking was found to positively predict total drinks consumed, however, there were no interaction effects between the BIS/BAS subscales and affect. These findings challenge existing affect regulation models and have implications for interventions aimed at addressing hazardous drinking.

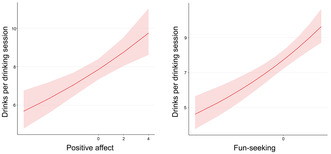

## INTRODUCTION

Epidemiological evidence has consistently revealed a high prevalence of problematic alcohol consumption in young adults. In large‐scale national surveys, approximately 30%–40% of young adults in the United States (Substance Abuse and Mental Health Services Administration, 2021) and similar proportions across Australia, Europe, and New Zealand (Australian Institute of Health and Welfare, 2020; Bartoli et al., 2014; Kypri et al., 2009) have been found to regularly engage in high‐risk drinking (i.e., more than four standard drinks in a day). Heavy patterns of consumption are associated with a plethora of deleterious health and social consequences, including assault and aggressive behavior, motor vehicle accidents, neural and cognitive impairments, and vulnerability to alcohol dependence (Jennison, 2004; Jones et al., 2018; Lees et al., 2019). While a considerable body of literature has examined the acute and long‐term effects of hazardous alcohol consumption, there has been a growing interest in the antecedents of alcohol use over the past two decades. Given the risk of compromised long‐term health outcomes associated with heavy patterns of consumption, it is imperative to investigate the mechanisms underlying hazardous alcohol use in order to provide targets for interventions.

Theoretical models posit that affect may be critical for understanding the processes underpinning high‐risk alcohol consumption (Wray et al., 2014). That is, individuals may consume alcohol either to ameliorate negative emotions or to heighten positive emotional states (Cooper et al., 1995; Cox & Klinger, 1988; Herman & Duka, 2019). This is problematic as it can lead to the consumption of higher quantities of alcohol and may also reinforce alcohol‐seeking behaviors (Patrick et al., 2011). Surprisingly, however, findings from both cross‐sectional and micro‐longitudinal studies have been equivocal. For example, several studies employing ecological momentary assessment or daily diary approaches have found that both daytime positive and negative affect predict alcohol use later that evening in young adults (Armeli et al., 2005; Bresin & Fairbairn, 2019; Dvorak et al., 2014, 2016; Dvorak & Simons, 2014; Russell et al., 2020; Simons et al., 2010, 2014). These studies largely indicate that increased affect is associated with greater consumption; however, the evidence appears to be more consistent for positive affect. Indeed, a number of studies have found no effect of daily negative affect on drinking (Crooke et al., 2013; Dvorak et al., 2018; Peacock et al., 2015). In concert with these findings, a recent meta‐analysis of individual‐level data found that individuals are more likely to initiate alcohol consumption and consume heavy quantities on days where they experience high positive affect rather than negative affect (Dora, Piccirillo, et al., 2023). Taken together, the literature suggests that individuals are more likely to initiate consumption and drink more heavily on days characterized by heightened positive affect, while negative affect does not appear to predict drinking on the daily level. It is important, however, to consider the influence of factors that may determine an individual's tendency to partake in excessive consumption under positive and negative circumstances.

There is a general consensus that higher levels of impulsivity are associated with greater alcohol use and related problems in young adults (Adams et al., 2013; Caswell et al., 2016; Henges & Marczinski, 2012; Lannoy et al., 2017). While definitions of impulsivity vary widely, impulsivity typically refers to the propensity to act quickly, without forethought or consideration of negative consequences (Evenden, 1999). The relationship between affect and impulsivity is important to consider as individuals high in impulsivity are purported to be more likely to act rashly in response to negative and positive emotions (Cyders et al., 2007). Indeed, previous work has demonstrated that individuals reporting higher levels of fun‐seeking are more likely to consume greater quantities of alcohol, and that this relationship is partially mediated by enhancement motives (Studer et al., 2016). On a daily level, there are mixed findings regarding the moderating role of impulsivity on affect‐driven consumption. For example, trait urgency (i.e., the propensity to act impulsively in response to positive and negative emotions) has been found to predict drinking to intoxication following increased affect (Bold et al., 2017; Simons et al., 2010). Conversely, Dora et al. (2022) found no effect of baseline or momentary negative urgency and negative affect on alcohol use, while trait positive urgency was found to interact with positive affect to predict subsequent alcohol use. In a pilot study, Stamates et al. (2019) measured momentary trait impulsivity in a small sample and found that positive affect moderated the relationship between impulsivity and alcohol‐related problems but not total number of drinks. Other studies have used a baseline measure of behavioral impulsivity (i.e., response inhibition) to predict drinking on a daily level and have found that positive mood predicted alcohol‐related problems in participants with poorer response inhibition (Dvorak et al., 2016). Given the sparsity of research, however, further work is necessary to clarify the role of other measures of impulsivity in affect‐driven consumption.

To date, findings on the role of positive and negative affect in daily alcohol consumption suggest that positive affect is more strongly associated with daily alcohol use in young adults. Moreover, the effect of trait impulsivity on daily affect and alcohol consumption has been largely unexamined. Understanding the role of affect on consumption is critical for informing targeted interventions to minimize alcohol‐related harm. Therefore, the purpose of this study was to employ a large sample of young adults to clarify whether positive and/or negative affect predict total consumption on drinking days and whether facets of impulsivity moderate these relationships, as measured by the Behavioral Inhibition System/Behavioral Activation Systems (BIS/BAS; Carver & White, 1994) scales. We focus on drinking days in order to determine whether affect and impulsivity predict greater alcohol intake during active drinking events. Previous work has demonstrated that individuals high on BAS (drive, reward responsiveness, and fun‐seeking) display sensation‐seeking tendencies and respond with increased positive affect to reward‐related cues (Wardell et al., 2011). Conversely, high BIS (i.e., motivation to avoid aversive outcomes) has been found to be related to anxiety and negative affect and has been proposed to be associated with problematic consumption due to negative reinforcement mechanisms (O'Connor & Colder, 2005; Smillie et al., 2006). Thus, we hypothesized that the BAS subscales (drive, reward responsiveness, and fun‐seeking) would interact with positive affect to predict total drinks consumed on drinking days, while the BIS subscale would moderate the relationship between negative affect and total drinks.

## MATERIALS AND METHODS

### Participants

The current study was conducted as part of the broader micro‐longitudinal Daily Life study which examined the biological and genetic markers of well‐being in young adults living in Dunedin, New Zealand (Conner et al., 2015). Participants were recruited through the University of Otago's psychology participation pool or through postings on online job boards. Participants were required to be aged 18–25 years, have a personal mobile phone and regular access to the Internet. All participants provided written informed consent for their involvement and the study was approved by the University of Otago Human Ethics Committee (10/777). Psychology students were reimbursed with course credit and those recruited elsewhere were provided remuneration. All reimbursement was scaled based on the level of participation. Of the original 1482 people who consented and completed the initial survey, 1416 completed the initial survey and the minimum number of daily diaries (7 of 13 diaries) and texting protocol (half of texts). As we were interested in regular drinkers, our sample only included participants who reported drinking on at least 2 days over the 13‐day study period. The final sample used in analysis comprised 693 participants (*M*_age_ = 19.71 years, SD = 2.04; female = 62.9%). A set of power simulations (1000 iterations) were conducted using code adapted from Dora, Mccabe, et al. (2023) to determine the power afforded by our sample to detect the smallest effect of interest (OR = 1.05). These simulations showed that with this sample size, power = 0.95.

### Measures

#### Behavioral Inhibition/Behavioral Activation Systems Scales (BIS/BAS)

The BIS/BAS scale (Carver & White, 1994) is a self‐report questionnaire that assesses an individual's sensitivity to punishment and reward. The measure includes a BIS subscale and three BAS subscales (drive, reward responsiveness, and fun‐seeking). Participants indicate their agreement to statements such as “I worry about making mistakes” and “I enjoy trying new things” using a 4‐point Likert scale. Higher scores on the BIS subscale indicate greater sensitivity for avoidance behaviors, while higher scores on the BAS subscale reflect a greater propensity for approach behaviors and impulsiveness.

#### Affect

To assess momentary affect, participants were delivered a text message that stated: “RIGHT NOW: How are you feeling? (1) Motivated (2) Positive (3) Energetic (4) Tense (5) Negative. 1 not at all – 9 extremely”. Participants responded by entering a single text with five numbers (e.g., 56,321), where each number represented a mood term rated on a scale from 1 to 9 respectively. The 1–9 response scale was selected to maximize variability and the numbers aligned with feature phone keypads used at the time. Motivated was asked to indicate approach motivation. The positive state question was asked to assess current positive valence and the negative state question was asked to assess current negative valence (Schimmack & Grob, 2000; Wilhelm & Schoebi, 2007). Energetic and tense measured energetic arousal and tense arousal specifically (Schimmack & Grob, 2000; Wilhelm & Schoebi, 2007). A research assistant explained each of the items of the question to ensure all participants understood the meaning of the text items. For the purposes of this study, only the “positive” and “negative” items were used in analysis as these items capture positive valence and negative valence in momentary affect. The “positive” and “negative” items demonstrated high internal reliability across time ($R_{{\mathrm{kRn}}_{\text{Positive}}}$ = 0.95; $R_{{\mathrm{kRn}}_{\text{Negative}}}$ = 0.95) (Revelle & Condon, 2019).

#### Alcohol

Participants recorded the number of drinks they consumed the previous night with one serving representing one standard drink (10 g of pure alcohol). Specifically, participants were asked, “How many standard drinks of alcohol did you have last night? (i.e., after you completed yesterday's survey) [or after 6:00 pm yesterday if this is your first day of the survey] until you went to sleep?” A standard drink guide for a range of commonly consumed beverages was provided to assist participants in their estimations. Participants were asked to enter “0” on days where no alcohol was consumed. Values were rounded to the nearest integer. Participants were also asked to provide the start time of the drinking session. Alcohol use was reverse lagged so that it aligned with daily assessments of positive and negative affect. All affect questions that were answered after drinking (and thus influenced by intoxication) were excluded from analyses.

### Procedure

Participants completed an initial baseline session which involved providing written informed consent, completing an online demographic and trait survey (including the BIS/BAS), and undertaking training on how to engage with the online survey platform. Following the baseline session, participants undertook a 13‐day online daily diary and text‐messaging protocol. The daily diary was administered between 3 and 8 pm and comprised a range of questions regarding recent experiences, including the number of standard drinks consumed the previous night. For the text‐messaging protocol, participants received four identical text messages per day to rate their current affect states using five items. The texts were delivered at semi‐random times within four equal intervals across the day, spaced at least 30 min apart. Texts were scheduled between 9 am and 9 pm on weekdays (Monday to Friday) and 12 pm and 10 pm on weekends (Saturday and Sunday). Response rates to the diary and texting protocol were tracked throughout the 13‐day period. Participants were provided with encouragement and additional text reminders to maintain compliance. Participants completed an in‐person follow‐up session at the conclusion of the study where they were debriefed and remunerated for their participation.

### Data analysis

Data analysis was undertaken using the programming language R (R Core Team, 2017). The resulting data had a multilevel structure such that observation days were nested within participants. Therefore, to assess the influence of affect and impulsivity on total drinks consumed on drinking days, we computed Generalized Linear Mixed Models (GLMMs) using the *glmmTMB* package (Brooks et al., 2017). Random intercept models were specified with participant ID as a random effect. Given that our outcome (total drinks) was an over‐dispersed count variable, data were modeled using a negative binomial distribution. Consistent with previous work, separate GLMMs were computed for positive and negative affect (e.g., Dora, Piccirillo, et al., 2023) and the best fitting models were based on evaluation of the AIC and BIC. Total drinks consumed was predicted by affect directly preceding the start time of the drinking session, BIS/BAS subscale scores (drive, reward responsiveness, fun‐seeking, and BIS), and the interaction between affect and BIS/BAS scores. Gender and total drinking days across the study period were included as covariates. Consistent with previous work, measures of positive and negative affect were person‐mean centered and standardized to *z*‐scores such that each participant's daily affect scores reflected deviations from their own mean (e.g., Dora, Piccirillo, et al., 2023). Since total drinking days and BIS/BAS scores were between‐subject variables (i.e., only one score was available per person), these variables were grand‐mean centered and standardized such that each participant's score reflected deviations from the mean of the sample. Incidence rate ratios (IRRs) were used to interpret our hypothesized associations. IRRs estimate the change in the incidence rate of a count measure (i.e., total drinks consumed) associated with a change in a continuous measure (i.e., affect and impulsivity measures). Given we were only interested in two estimated effects (i.e., the main effect of affect and the interaction between affect and BIS/BAS subscales), a Bonferroni‐adjusted family‐wise alpha of 0.010 was assumed for our primary analyses. Participants with minimum data were included as multilevel models are robust to missing data. Further, since we only included days where alcohol was consumed, missing data were minimal (2.8%). For completeness, we also report models that use aggregated affect in place of lagged affect. That is, positive and negative affect scores were each averaged using all prompts that preceded the drinking session (see Appendix S1).

## RESULTS

### Descriptive statistics

Descriptive statistics are displayed in Table 1. Over the 13‐day study period, participants completed an average of 11.53 surveys (SD = 1.49; range = 5–13) and 48.59 text‐message prompts (SD = 4.86; range = 6–52). On average, participants were found to consume alcohol on 3.06 days (SD = 1.33). The average number of drinks consumed per drinking day was 7.20 (SD = 5.74). Average pre‐consumption positive affect (*M* = 6.68, SD = 1.74) was higher than pre‐consumption negative affect (*M* = 2.09, SD = 1.50).

**TABLE 1:** Descriptive statistics of sample characteristics.

	*n* (%)	Mean (SD)
Females	436 (62.9)	
Year at university		
First year	242 (34.9)	
Second year	232 (33.5)	
Third year	117 (16.9)	
Fourth year	63 (9.1)	
Post‐graduate	39 (5.6)	
Ethnicity		
European	613 (88.5)	
Asian	34 (4.9)	
Māori/Pacific Islander	28 (4.0)	
Other	18 (2.6)	
Completed daily surveys		11.53 (1.49)
Completed text‐message prompts		48.59 (4.86)
Total drinking days		3.06 (1.33)
Total drinks per drinking day		7.20 (5.74)
Total drinks over 13 days		21.57 (16.22)
Pre‐consumption aggregated positive affect		6.59 (1.48)
Pre‐consumption aggregated negative affect		2.13 (1.25)
Pre‐consumption positive affect		6.68 (1.74)
Pre‐consumption negative affect		2.09 (1.50)
Drive		11.03 (2.03)
Fun‐seeking		12.83 (1.95)
Reward		17.12 (1.86)
BIS		20.95 (3.64)
BAS		40.98 (4.43)

Abbreviations: BAS, Behavioral Activation System; BIS, Behavioral Inhibition System; SD, standard deviation.

### Generalized linear mixed models

Table 2 displays the results of the GLMM predicting total drinks consumed from positive affect and BIS/BAS subscales. Results indicated a small main effect of pre‐consumption positive affect on alcohol consumption, IRR = 1.06, 95% CI [1.03, 1.09], such that higher pre‐consumption positive affect predicted a greater number of drinks consumed. Specifically, a one‐point increase in positive affect predicted approximately an additional half a drink consumed per drinking session. Further, fun‐seeking was found to positively predict total drinks, IRR = 1.14, 95% CI [1.09, 1.19]. There was, however, no significant interaction effect between positive affect and fun‐seeking, nor any other BIS/BAS subscale.

**TABLE 2:** Generalized linear mixed model predicting total drinks consumed from pre‐consumption positive affect and BIS/BAS subscales.

Predictors	Incidence rate ratios	CI	*p*
(Intercept)	8.41	7.79–9.08	**<0.001**
Total drinking days	0.91	0.87–0.95	**<0.001**
Sex [female]	0.73	0.67–0.80	**<0.001**
Drive	0.99	0.94–1.04	0.668
Reward	0.99	0.94–1.04	0.640
Fun‐seeking	1.14	1.09–1.19	**<0.001**
BIS	0.99	0.95–1.04	0.698
Pre‐consumption PA	1.06	1.03–1.09	**<0.001**
Pre‐consumption PA × Drive	1.00	0.97–1.03	0.850
Pre‐consumption PA × Reward	1.02	0.99–1.05	0.111
Pre‐consumption PA × Fun‐seeking	0.98	0.95–1.00	0.079
Pre‐consumption PA × BIS	0.99	0.97–1.02	0.614
Random effects			
*σ* ^2^	0.37		
*τ*_00 ID_	0.15		
ICC	0.29		
*N*_ID_	693		
Observations	2033		
Marginal *R* ^2^/Conditional *R* ^2^	0.080/0.343		

*Note*: Total drinking days and BIS/BAS subscales were grand‐mean centered. Positive affect was person‐mean centered. Bold values indicate statistically significant effects.

Abbreviations: BAS, Behavioral Activation System; BIS, Behavioral Inhibition System; CI, confidence interval; ICC, intraclass correlation; PA, positive affect.

Results of the GLMM predicting total drinks consumed from negative affect and BIS/BAS subscales are displayed in Table 3. There was no main effect of pre‐consumption negative affect on alcohol consumption, IRR = 0.98, 95% CI [0.96, 1.01]. While there was a trend for an interaction between negative affect and the BIS subscale, no significant interaction effects were found between negative affect and BIS/BAS subscales. Results were the same for models predicting total drinks from aggregated daily affect (see Appendix S1).

**TABLE 3:** Generalized linear mixed model predicting total drinks consumed from pre‐consumption negative affect and BIS/BAS subscales.

Predictors	Incidence rate ratios	CI	*p*
(Intercept)	8.44	7.82–9.11	**<0.001**
Total drinking days	0.91	0.87–0.95	**<0.001**
Sex [female]	0.73	0.67–0.80	**<0.001**
Drive	0.99	0.94–1.04	0.674
Reward	0.99	0.94–1.04	0.640
Fun‐seeking	1.14	1.08–1.19	**<0.001**
BIS	0.99	0.95–1.03	0.666
Pre‐consumption NA	0.98	0.96–1.01	0.236
Pre‐consumption NA × Drive	0.99	0.96–1.02	0.479
Pre‐consumption NA × Reward	1.01	0.99–1.04	0.343
Pre‐consumption NA × Fun‐seeking	1.01	0.98–1.04	0.447
Pre‐consumption NA × BIS	1.02	0.99–1.05	0.195
Random effects			
*σ* ^2^	0.37		
*τ*_00 ID_	0.14		
ICC	0.28		
*N*_ID_	693		
Observations	2033		
Marginal *R* ^2^/Conditional *R* ^2^	0.075/0.334		

*Note*: Total drinking days and BIS/BAS subscales were grand‐mean centered. Negative affect was person‐mean centered. Bold values indicate statistically significant effects.

Abbreviations: BAS, Behavioral Activation System; BIS, Behavioral Inhibition System; CI, confidence interval; ICC, intraclass correlation; NA, negative affect.

## DISCUSSION

Using a large sample of young adults, the present study adopted an ecological momentary assessment design to determine whether trait impulsivity—measured using the BIS/BAS—moderates the relationship between daily affect and alcohol consumption. The results of the study demonstrate that participants were more likely to drink in greater quantities on drinking occasions preceded by higher positive affect but not negative affect. While fun‐seeking was found to positively predict total drinks consumed, there were no interaction effects between the BIS/BAS subscales and affect on alcohol consumption. These findings add to existing work and suggest the need to reconsider models of affect regulation, particularly in young adults.

Our finding that higher positive affect predicted an increased likelihood of greater consumption is consistent with a large body of work (Bresin & Fairbairn, 2019; Dora, Piccirillo, et al., 2023; Dvorak et al., 2014, 2016; Dvorak & Simons, 2014; Russell et al., 2020; Simons et al., 2010, 2014). Research has shown that induction of positive mood increases feelings of sociability and a preference for seeking out social situations (Whelan & Zelenski, 2012). Therefore, positive affect may drive drinking behavior by triggering individuals to partake in social activities where alcohol may be accessible (Dora, Piccirillo, et al., 2023). It is also plausible that individuals expect to feel positive following alcohol use and are thus experiencing increased positive affect in anticipation of drinking (Dvorak et al., 2018). It should be noted, however, that the current design does not permit us to draw conclusions on the reasons behind why positive affect may be driving drinking behavior. Thus, further work is required to clarify the role of the drinking motive and context in affect‐driven consumption.

While our finding of no significant effect of negative affect on total drinks consumed is in keeping with previous work, it is nonetheless surprising given that models of affect regulation have consistently argued that people drink more to ameliorate negative emotions (Cooper et al., 1995). Considering our results together with recent research, it appears that young adults do not necessarily consume more alcohol on days characterized by heightened negative affect. Given recent work, it is plausible that relative changes in negative affect might influence the motivation underlying drinking (e.g., increased coping motivation) rather than the amount consumed. Nonetheless, it is important to consider the role of social contexts, particularly given that the current sample largely comprised first‐ and second‐year undergraduate students. Research has demonstrated that student drinking typically occurs in social or celebratory contexts (Baer, 2002; Glindemann et al., 2007), and is thus likely to be preceded by positive affect rather than negative affect (Howard et al., 2015). It should be noted that several previous studies which have discerned an effect of heightened negative mood on increased consumption have involved non‐student populations. For example, Duif et al. (2020) and Russell et al. (2020) found a significant effect of negative affect in community samples with mean ages higher than the current sample (*M*_age_ = 36.07 years, SD = 9.23, and *M*_age_ = 23.4 years, SD = 7.4, respectively). Other studies have reported an effect of negative affect on drinking in samples that exhibit acute alcohol use disorder symptoms or heavy drinking (Armeli et al., 2000; Dvorak et al., 2014). The drinking patterns of these samples are likely to differ substantially from those of student samples. Thus, it remains plausible that heightened negative affect is related to increased drinking in individuals who exhibit clinically relevant levels of consumption or who tend to drink predominantly outside of social contexts.

Contrary to expectations, we did not find evidence in support of an interaction between affect and impulsivity in predicting total drinks consumed. Extant work has demonstrated that impulsivity is positively associated with substance misuse, including heavy alcohol consumption, in college samples (Adams et al., 2013; Caswell et al., 2016; Murphy & Garavan, 2011). Previously, it has been proposed that highly impulsive individuals have a greater tendency for reacting to positive and negative emotions (Dick et al., 2010). While we found evidence that fun‐seeking was associated with greater alcohol use, there was no effect of fun‐seeking—or other BIS/BAS subscales—on the relationship between affect and alcohol use. It is worth acknowledging that our finding of a relationship between fun‐seeking and consumption is consistent with a large body of work. In fact, most studies employing the BIS/BAS scale in investigations of alcohol use have reported significant positive associations between fun‐seeking and alcohol use (Franken & Muris, 2006; O'Connor et al., 2009; Studer et al., 2016; Yen et al., 2009). While our results do not support our initial hypotheses regarding an interaction effect, they do not discount the possibility that other facets of impulsivity may moderate the relationship between affect and total consumption. For example, there is some evidence that trait urgency predicts greater affect‐related consumption, particularly for days higher in positive affect (Bold et al., 2017; Dora et al., 2022). The urgency subscales of the UPPS‐P used in these studies specifically capture the propensity to respond impulsively to positive and negative emotions (Whiteside & Lynam, 2001). It is thus likely that measures of urgency are more closely associated with daily affect‐driven consumption than the BIS/BAS scale used in the current study.

A major strength of the current study is that it comprises one of the largest samples to date in the investigation of daily affect and alcohol consumption. Further, this study is one of few to examine the role of impulsivity in the relationship between daily affect and consumption. These results have implications for the development of future prevention initiatives, particularly targeting young adults using ecological momentary interventions. Previous work has demonstrated that, in young adults, an additional drink per drinking session doubles the number of consequences experienced (Barnett et al., 2014). The current results highlight affect as a potential target for future interventions. Specifically, detecting increased positive affect in young adults may provide the opportunity to interrupt excessive drinking trajectories before they are initiated.

There are, however, limitations that should be considered. First, the current design did not permit an analysis of post‐consumption changes in affect. To determine change in affect following alcohol use, an assessment of affect after consumption, and ideally after resolution of any intoxication, is required. Participants in the current study were prompted for four daily assessments of affect and were found to typically initiate alcohol use by the final assessment or thereafter. Thus, a post‐consumption affect assessment was unavailable for a large proportion of drinking sessions. A further limitation is that we only acquired a baseline assessment of impulsivity and therefore were unable to link daily fluctuations in impulsivity to affect and consumption. Previous work has demonstrated that daily changes in impulsivity are associated with alcohol use (Trull et al., 2016). It is largely unknown, however, whether these fluctuations interact with changes in affect to predict consumption (although see Stamates et al., 2019 for preliminary findings). Future work should seek to clarify the role of both trait and state impulsivity in moderating the relationship between affect and alcohol use on a momentary level.

The current study investigated the role of impulsivity in affect‐related alcohol use in a large sample of young adults. Our results demonstrate that positive affect, but not negative affect, predicts alcohol consumption such that higher positive affect is related to greater consumption. While we discerned evidence of a relationship between fun‐seeking and increased alcohol use, there was no interaction effect between impulsivity and affect. These results challenge affect regulation models and present implications for the development of ecological momentary interventions targeting hazardous drinking.

## FUNDING INFORMATION

This research was supported by a University of Otago Research grant and a Health Research Council of New Zealand Grant (12/709) to T.S.C. This research was also supported by a Synergy grant from the National Health and Medical Research Council (2021/GNT200985) to P.S.H., K.C.M., G.P.M., V.M., and E.Z.M.

## CONFLICT OF INTEREST STATEMENT

None.

## Supporting information


Appendix S1
