# Supplementary material for: The role of impulsivity in the relationship between affect and alcohol consumption in young adults
Source: Alcohol Clin Exp Res (Hoboken). 2023 Sep 24;47(11):2161–8. doi: 10.1111/acer.15192 (PMC10946841; doi:10.1111/acer.15192)
Supplement: Supplementary file 1 — Appendix S1 [file ACER-47-2161-s001.docx]

**Supplementary Material**

Tables S1 and S2 display results for the generalized linear mixed models predicting total drinks from aggregated positive and negative affect and BIS/BAS subscales. Positive and negative affect scores were each averaged using all prompts that preceded the drinking session to create an aggregated value for each drinking day. For example, if drinking occurred at 19:00 and the participant reported affect scores at 12:00, 15:00, 18:00 and 20:00, the average of affect scores at 12:00, 15:00 and 18:00 was matched to drinking at 19:00 for that day. The analyses reported in the main manuscript use only the affect scores most closely preceding consumption. In this example, only the positive and negative affect scores reported at 18:00 would have been matched to drinking at 19:00.

**Table S1**

*Generalized Linear Mixed Model Predicting Total Drinks from Aggregated Positive Affect and BIS/BAS Subscales*

| *Predictors* | *Incidence Rate Ratios* | *CI* | *p* |
| --- | --- | --- | --- |
| (Intercept) | 8.32 | 7.71 – 8.98 | **<0.001** |
| Total drinking days | 0.91 | 0.87 – 0.95 | **<0.001** |
| Sex [female] | 0.72 | 0.65 – 0.78 | **<0.001** |
| Drive | 0.99 | 0.95 – 1.04 | 0.769 |
| Reward | 0.99 | 0.94 – 1.04 | 0.606 |
| Fun-seeking | 1.14 | 1.08 – 1.19 | **<0.001** |
| BIS | 0.99 | 0.95 – 1.04 | 0.721 |
| Daily PA | 1.09 | 1.06 – 1.12 | **<0.001** |
| Daily PA × Drive | 0.99 | 0.96 – 1.02 | 0.577 |
| Daily PA × Reward | 1.03 | 1.00 – 1.06 | 0.093 |
| Daily PA × Fun-seeking | 0.98 | 0.95 – 1.01 | 0.273 |
| Daily PA × BIS | 0.99 | 0.96 – 1.02 | 0.514 |
| **Random Effects** | | | |
| σ^2^ | 0.37 | | |
| τ_00_ _ID_ | 0.14 | | |
| ICC | 0.28 | | |
| N _ID_ | 693 | | |
| Observations | 2033 | | |
| Marginal R^2^ / Conditional R^2^ | 0.089 / 0.342 | | |

*Note.* Total drinking days and BIS/BAS subscales were grand-mean centered. Positive affect was person-mean centered. BAS = Behavioral Activation System; BIS = Behavioral Inhibition System; CI = confidence interval; ICC = intraclass correlation; PA = positive affect.

**Table S2**

*Generalized Linear Mixed Model Predicting Total Drinks from Aggregated Negative Affect and BIS/BAS Subscales*

| *Predictors* | *Incidence Rate Ratios* | *CI* | *p* |
| --- | --- | --- | --- |
| (Intercept) | 8.46 | 7.84 – 9.13 | **<0.001** |
| Total drinking days | 0.91 | 0.87 – 0.95 | **<0.001** |
| Sex [female] | 0.73 | 0.66 – 0.79 | **<0.001** |
| Drive | 0.99 | 0.94 – 1.04 | 0.654 |
| Reward | 0.99 | 0.95 – 1.04 | 0.712 |
| Fun-seeking | 1.14 | 1.08 – 1.19 | **<0.001** |
| BIS | 0.99 | 0.95 – 1.04 | 0.713 |
| Daily NA | 0.98 | 0.95 – 1.01 | 0.145 |
| Daily NA × Drive | 1.01 | 0.98 – 1.04 | 0.657 |
| Daily NA × Reward | 1.01 | 0.98 – 1.04 | 0.697 |
| Daily NA × Fun-seeking | 1.00 | 0.97 – 1.03 | 0.963 |
| Daily NA × BIS | 1.02 | 0.99 – 1.05 | 0.260 |
| **Random Effects** | | | |
| σ^2^ | 0.37 | | |
| τ_00_ _ID_ | 0.14 | | |
| ICC | 0.28 | | |
| N _ID_ | 693 | | |
| Observations | 2033 | | |
| Marginal R^2^ / Conditional R^2^ | 0.074 / 0.333 | | |

*Note.* Total drinking days and BIS/BAS subscales were grand-mean centered. Negative affect was person-mean centered. BAS = Behavioral Activation System; BIS = Behavioral Inhibition System; CI = confidence interval; ICC = intraclass correlation; NA = negative affect.
